# Inconformity of CXCL3 Plasma Level and Placenta Expression in Preeclampsia and Its Effect on Trophoblast Viability and Invasion

**DOI:** 10.1371/journal.pone.0114408

**Published:** 2014-12-08

**Authors:** Shunping Gui, Shanshan Ni, Jin Jia, Yunhui Gong, Linbo Gao, Lin Zhang, Rong Zhou

**Affiliations:** 1 Department of Obstetrics and Gynaecology, West China Second University Hospital, Sichuan University, Chengdu, P.R.China; 2 Key Laboratory of Obstetric & Gynecologic and Pediatric Diseases and Birth Defects of Ministry of Education, Chengdu, P.R.China; 3 Laboratory of Molecular and Translational Medicine, West China Institute of Women and Children's Health, West China Second University Hospital, Chengdu, P.R.China; Medical Faculty, Otto-von-Guericke University Magdeburg, Medical Faculty, Germany

## Abstract

As a member of the chemokine family, CXCL3 was previously known to participate in many pathophysiological events. However, whether CXCL3 stimulates trophoblast invasion as a key process of preeclampsia pathogenesis remains largely unknown. Therefore, the aim of this study was to investigate this hypothesis and determine the effect of CXCL3 on the first trimester trophoblast. Seventy gravidas were included in this study. ELISA was used to detect CXCL3 plasma levels on preeclampsia and normal pregnant groups. CXCL3 protein and mRNA levels were detected via Western blot and real-time quantitative PCR analysis after immunolocalized in human placenta. Moreover, the CXCL3 function in HTR-8/Svneo was analyzed via WST-1 assay, flow cytometry and invasion test. The plasma CXCL3 level in preeclampsia was significantly higher than that in normal pregnancy. CXCL3 expression was observed in the cytoplasm of placental trophoblasts and vascular endothelium in all groups without significant difference between maternal and fetal sides. In addition, placenta CXCL3 expression in severe preeclampsia was significantly lower than those in normal and mild PE groups. Moreover, exogenous CXCL3 can promote the proliferation and invasion of HTR-8/Svneo; however, its effect on apoptosis remains unclear. In summary, a significant abnormality of plasma CXCL3 level and placental CXCL3 expression was discovered in severe preeclampsia; CXCL3 had a function in trophoblast invasion, which indicated its participation in shallow implantation. Therefore CXCL3 might be involved in severe preeclampsia pathogenesis.

## Introduction

Preeclampsia is an important and specific complication of pregnancy. This condition is characterized by the onset of hypertension after 20 weeks of gestation with accompanying proteinuria, which affects approximately 3.4% of all pregnancies [1]. Preeclampsia is a major cause of maternal mortality and a risk factor for fetal growth restriction and fetal death [2]. Increasing evidence have suggested that preeclampsia result from placental dysfunction during the first trimester, which is caused by the shallow invasion of extravillous trophoblast cells and impaired vascular development in the placental bed [3], [4].

Chemokines function as regulators of leukocyte migrate to damaged tissue site when inflammation or infection occurs. Chemokines are divided into four subfamilies (namely, CXC, CC, C, and CX3C) based on different conserved cysteine residues parts. As a part of CXC chemokines, growth-related oncogene (GRO) chemokine is encoded by the human GRO gene. GRO is divided into three extremely homologous subtypes, namely, GRO-α (CXCL1), GRO-β (CXCL2), and GRO-γ (CXCL3) [5]. CXCL3 (GRO-γ) belongs to the CXC family bearing(ELR+) motif, glutamic acid(E), leucine(L), and arginie(R). And the ELR+ motif precedes the first two cysteines, which are separated by one amino acid (CXC) [6], [7]. CXCL3 functions after binding the receptor, CXCR2. CXCR2 promotes chemotaxis and angiogenesis via the extracellular signal-regulated protein kinase 1/2 (ERK 1/2) pathway after combining with ELR+ CXC chemokine. CXCR2 is expressed in human vascular endothelial cells, basophilic granulocytes, and T lymphocytes [8], [9]. One recent study had demonstrated that CXCL3 is involved in precursors of cerebellar granule neuron migration [10].

Chemokines affect angiogenesis and have a function in cell migration and invasion. Studies have confirmed that several subtypes of chemokines (namely, CX3CL1, CCL14, and CCL4) can enhance the migration and invasion ability of trophoblast cell, whereas the presence of trophoblast cells can increase the amount of chemokines (namely, CXCL1, CXCL2, CXCL10, CCL8, and receptor CXCR4) [11], [12], [13], [14]. However, Zhang's study demonstrated that chemokine CXCL6 restricts human trophoblast cell migration and invasion [15]. CXCL3 also showed increased gene expression in response to GnRH agonist treatments in trophoblast cells, which may happen during the early stage of pregnancy [16]. Trophoblastic invasion to the endometrium and spiral artery remodeling are important processes during normal pregnancy. Moreover, failure of these processes to proceed can cause abnormal pregnancy complications, such as preeclampsia. Therefore, we presumed that CXCL3 was involved in preeclampsia pathogenesis. We compared CXCL3 plasma level and placental expression between the preeclampsia group and the normal pregnant group to determine the relationship between CXCL3 and preeclampsia. In particular, we investigated the contribution of CXCL3 to the biological behavior of trophoblast cells.

## Materials and Methods

### Plasma and tissue specimens

Seventy gravidas who were referred to the obstetrics unit of West China Second University Hospital from April 2011 to December 2011 were included in this study. Preeclampsia is characterized by hypertension (systolic blood pressure ≥140 mmHg and diastolic blood pressure ≥90 mmHg after 20 week gestation) and proteinuria (≥300 mg in a 24 hr urine collection or one dipstick measurement ≥1+). This criterion was based on the recommendations of the American College of Obstetricians and Gynecologists [17]. Severe preeclampsia was diagnosed based on diastolic blood pressure ≥110 mmHg or significant proteinuria (dipstick measurement of ≥2+) or the presence of severity symptoms, such as headache, visual disturbances, upper abdominal pain, oliguria, convulsion, elevated serum creatinine, thrombocytopenia, marked liver enzyme elevation, and pulmonary edema. A total of 45 confirmed cases of preeclampsia were included in the study, 20 of which were mild preeclampsia(mild PE group) and the other 25 were severe preeclampsia(severe PE group). A total of 25 healthy control subjects were also included in the study for comparison. All pregnant women were dated according to routine ultrasound measurements in the first trimester, and preterm birth was defined as <37 and 0/7 completed weeks of gestation. Written informed consent was obtained from all subjects, and the study was approved by the Institutional Ethics Committee of West China Second University Hospital. About 5 mL of blood was extracted from all patients with anticoagulant before labor and therapeutic intravenous administration of magnesium sulfate. Plasma was subsequently separated. All samples were stored at −80°C until further use.

Placental tissues were collected as described previously [18]. Placental tissue in the placenta central zone (i.e., umbilical cord attached to the contralateral maternal side) was collected immediately after operation from women undergoing elective Caesarean section without calcification and bleeding. All sealed samples were collected and stored at −80°C until required.

### Cell cultures

The extravillous trophoblast (EVT) cell line HTR-8/SVneo was provided by Dr Yali Hu of Nanjing Drum Tower Hospital, The Affiliated Hospital of Nanjing University Medical School, China. The said cell line was grown in RPMI 1640 (Hyclone, USA) supplemented with 10% heat-inactivated fetal calf serum (FCS), penicillin (100 U/mL), and streptomycin (100 µg/mL) at 37°C and 5% CO_2_.

### Enzyme-linked immunosorbent assay

CXCL3 plasma levels were determined using a commercial CXCL3 kit (Uscn Life Science Inc. China) by following the manufacturer's recommendations. All samples were run in duplicate, and the mean value was reported. The lower detection limit of CXCL3 in this assay was 7.8 pg/mL.

### Immunohistochemistry

Placental samples were fixed in formalin and embedded in paraffin. Sections (4 µm) were deparaffinized, rehydrated, treated with 4% hydrogen peroxide for 10 min in the dark at room temperature, and autoclaved at 95°C for 10 min in 0.05 mol/L Tris-EDTA solution (PH 9.0) before incubated with rabbit anti-human CXCL3 polyclonal antibody (1∶100 dilution, Bioss, China) at 37°C for 2 h. The sections were incubated with secondary antibody (goat anti-rabbit 1∶1,000; Beijing ZhongShan Biotechnology) for 45 min at 37°C after washing thrice in PBS. The sections were counterstained with hematoxylin and mounted with a cover glass. The negative control samples were incubated with PBS as an alternative for anti-human CXCL3 antibody. Colon cancer sections were used as positive control samples instead of placental tissue with routine staining.

### Western blotting

The 1∶1 mixture of 30 µg placenta samples and 2× loading buffer (2.5 mL 0.5 M Tris, pH 6.8, 2 mL glycerol, 2 mL 20% sodium dodecyl sulfate, and 3 mL double distilled water with bromphenol blue with a deep blue color) was boiled for 5 min before loading. The samples were separated on 15% sodium dodecyl sulfate–polyacrylamide resolving gels with a 5% stacking gel by using Mini-PROTEAN (Bio-Rad Laboratories, Inc., U.S.A.) at a constant voltage of 200 V. A prestained protein ladder (10 kDa to 250 kDa; Thermo Fisher Scientific, Lithuania) was loaded adjacent to the samples.

The protein was transferred for 25 min at a constant voltage of 90 V to Immobilon-P polyvinylidene difluoride (PVDF) membranes (0.45 µm, Merk Millipore, Germany) after pretreatment with methyl alcohol for 20 s. The absorbance of the membrane background was decreased by blocking this structure with 5% defatted milk for 30 min. Rabbit polyclonal anti-GRO gamma antibody (1∶100 dilution, Abcam, U.S.A.) and rabbit polyclonal anti-beta-actin (loading control, 1∶100 dilution, Bioss, China) were sealed up with the membranes at room temperature for 2 h and at 4°C overnight, respectively. The membranes were rinsed once and were subsequently washed twice for 1 min in TBST buffer (25 mM Tris, pH 7.5, 150 mM NaCl, 0.5% Tween). The membranes were then incubated with peroxidase-conjugated affiniPure goat anti-rabbit IgG (H+L, 1∶1,000, Beijing ZhongShan Biotechnology) with gentle shaking at room temperature for 1 h. Protein brands were detected using the CheniDoc XRS system (Bio-Rad Laboratories, Inc. U.S.A.) after washing. Each optical density was quantified using the Quantity One software.

### Real-time fluorescent quantitative PCR

Total RNA was extracted using a TRNzol-A^+^ reagent (Tiangen Biotech CO., Beijing, China), and its integrity was verified via agarose gel electrophoresis. Reverse transcription reactions were performed using the ReverTra Ace MMLV reverse transcriptase RnaseH- (Toyobo Co., Japan). Real-time PCR analyses were performed using Mastercycler epgradient S (Eppendorf, Germany) to determine the number of cDNA molecules in the reverse-transcribed samples. PCR was performed using 8.7 µL of 2× Maxima SYBR Green qPCR Master Mix (Fermentas Inc., Canada), 0.15 µL of each 5' and 3' primer, 1 µL of the cDNA samples, and 10 µL of H_2_O to a final volume of 20 µL. After the samples were denatured at 95°C for 10 min, amplification and fluorescence determination were performed in three steps, i.e., denaturation at 95°C for 15 s, annealing at 60°C for 1 min, and extension at 72°C for 30 s. Moreover, SYBR Green fluorescence was detected at the end of extension, and this value reflects the amount of double-stranded DNA. The amplification cycle number was 40. A melting curve was obtained at the end of each run to discriminate specific from nonspecific cDNA products. The data were normalized with GAPDH levels in the samples. The primer sequences used for real-time PCR were listed respectively as follows: forward: 5′-CGC CCA AAC CGA AGT CAT-3′, reverse: 5′-GTG CTC CCC TTG TTC AGT ATC T-3′ for the CXCL3; the primers for GAPDH (internal control) were: forward: 5′-GAA GGT GAA GGT CGG AGT C-3′, reverse: 5′-GAA GAT GGT GAT GGG ATT TC-3′ (synthesized by Beijing DNAchem Biotechnology Co., Ltd., China).

### Cell viability and apoptosis assay

HTR-8/SVneo cells, which were seeded into 96-well microtiter plates at 1×10^3^ cells/well concentration, were treated with Recombinant Human GRO-γ (rhCXCL3) at different concentrations and time gradients after 12 h of starvation. The proliferating activities of these cells were measured via water-soluble tetrazolium salt (WST-1) assay (Beyotime, China). About 10 µL of WST-1 was added per well. Moreover, the cells were incubated for 1 h before their optical density was analyzed using an Infinite 200 PRO microplate reader (Tecan Group Ltd., Switzerland) at 450 nm to determine cell viability. Each test was repeated in triplicate.

Apoptosis was assessed via staining by using annexin-V-conjugated fluorescein isothiocynate (FITC) and propidium iodide (PI) in an apoptosis detection kit (KeyGEN BioTECH, China). The cells were treated with different concentrations of recombinant proteins after being seeded on a slide for 24 h. Afterwards, the cells were washed with PBS twice and dyed in 500 µL of the binding buffer with 5 µL of Annexin V-FITC and 5 µL of PI for 5 min at room temperature. Finally, cell apoptosis quantification was performed using an inverted fluorescence microscope (Zeiss, Germany).

### Invasion assay

Cells that were treated with different concentrations of rhCXCL3 for 24 h after 12 h of starvation were suspended and seeded in the upper compartment of a Matrigel Matrix (diluented 1∶6; BD Biosciences, USA)-coated 24-well Transwell units (8 µm, Costar) without FCS. By contrast, 500 µL of media that contain 10% FCS was placed into the bottom chamber to function as a chemoattractant. After 48 h of incubation in a cell culture incubator, non-invaded cells were removed from the upper well by using cotton swabs. By contrast, the invaded cells, which invaded through the matrigel and 8 µm pores and subsequently adhered to the lower surface of the membrane, were fixed with 4% paraformaldehyde, stained with eosin, and photographed (×200) in five independent fields for each insert. Triplicate assays were performed for each group of cells. Cells that traversed the matrix and membrane were counted, and the total number of treated cells for each membrane was compared with untreated control samples.

### Statistical analyses

Data was expressed as mean ± standard deviation. All comparisons employed Student's t-test (paired, two-tailed) to compare the two groups. In addition, one-way ANOVA was used when more than two groups were compared. Spearman rank order correlation coefficients (continuous variables) and Mann–Whitney U-test (categorical variables) were performed to reveal the relationship between the results and clinical parameters. For cell viability assay results, repetitive measurement and ANOVA were adopted. The least significant difference (LSD) method was applied in analyzing multiple comparisons between groups, and P-value <0.05 was considered statistically significant.

## Results

### Baseline Characteristics

The characteristics of the participants are presented in Table 1. No significant difference was noted in maternal age and body mass index at early pregnancy among the three groups. Gestational age at sampling, gestational age at delivery, birth weight, and fetal length of severe PE group were significantly lower than the normal and mild PE group (P<0.05). By contrast, the four characteristics of preterm subgroup were significantly lower than that of full term subgroup in mild PE group (P<0.05). All pregnant women in the normal pregnant group underwent full-term delivery, and 16 out of 20 cases in mild preeclampsia group underwent full-term delivery. Moreover, all participants in the severe preeclampsia group were premature.

**Table 1:** Clinical characteristics of study participants.

	Normal pregnant group	Mild PE group		Severe PE group
	Full term(n = 25)	Full term(n = 16)	Preterm(n = 4)	Preterm(n = 25)
Maternal age (years)	31.00±5.60	30.88±6.47	26.75±5.12	30.00±5.30
Early pregnancy body mass index (kg/m^2^)	22.28±3.33	23.80±2.37	23.21±4.00	22.57±3.30
Gestational age at plasma sampling (weeks)	38.30±1.02	38.67±1.20	34.38±1.14☆	30.93±3.19△▴
Gestational age at delivery (weeks)	38.80±1.17	39.05±1.43	35.39±0.86☆	31.25±3.22△▴
Birth weight (g)	3362.00±440.50	3005.94±787.40	2386.25±528.79☆	1903.00±776.60△▴
Birth length (cm)	48.90±1.49	47.44±4.02	44.75±3.69☆	40.84±6.18△▴
24h urine protein	―	2.36±2.24	4.85±2.72	4.44±2.89
Mean arterial pressure	81.96±7.04	112.40±14.80	119.33±16.82	117.55±16.06

Data are presented as mean ± standard error of the mean.

☆:Full term pregnancy compared with preterm pregnancy in mild PE group, P<0.05;

△:Severe PE group compared with normal pregnant group, P<0.05;

▴:Severe PE group compared with mild PE group, P<0.05.

### Plasma CXCL3 concentration

**Figure 1:**
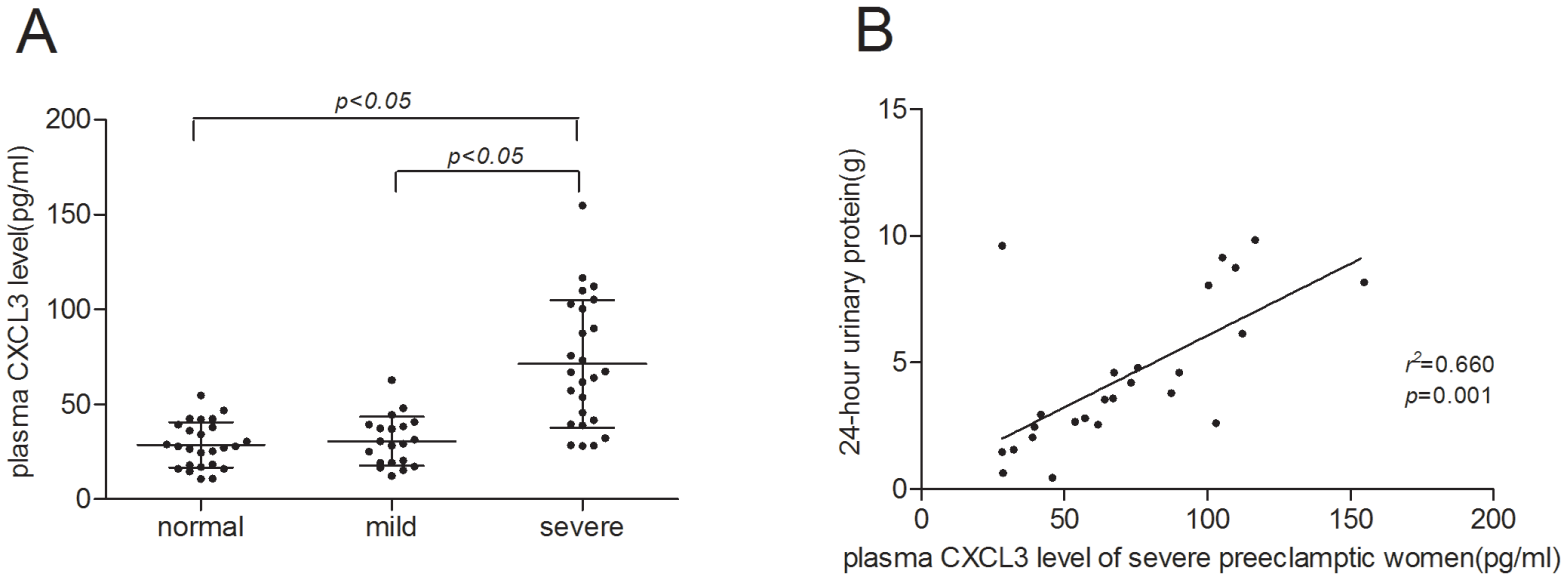
Plasma CXCL3 concentration. A:Plasma CXCL3 levels of normal pregnant women, mild preeclamptic pregnant women and severe preeclamptic pregnant women. Middle line: median; Whisker: standard deviation. B: The correlation between plasma CXCL3 levels of the severe PE group participants with 24-hour urinary protein.

Plasma CXCL3 level (71.31±33.65 pg/mL) in the severe preeclampsia group was significantly higher than that in the normal (28.71±11.91 pg/mL) and mild PE group (30.65±13.00 pg/mL) (P <0.05). However, the difference between the normal and mild PE group (P>0.05) was statistically insignificant. Termed pregnancy subgroup (31.50±12.86 pg/mL) was not significantly different from the preterm subgroup (27.26±14.98 pg/mL) in mild preeclamptic patients (P>0.05) (Fig. 1A).

We also investigated if the plasma CXCL3 levels of the study participants were related to their clinical features and laboratory parameters. The results showed that the plasma CXCL3 level of severe preeclampsia patients was positively correlated with 24 h urinary protein (r^2^ = 0.660) (Fig. 1B). However, no relationship between plasma CXCL3 level and antenatal blood pressure (as well as other indicators) was found. Plasma CXCL3 level is not correlated with all clinical features in mild patient group and normal pregnant group.

### Placental CXCL3 expression and CXCL3-mRNA expression

**Figure 2:**
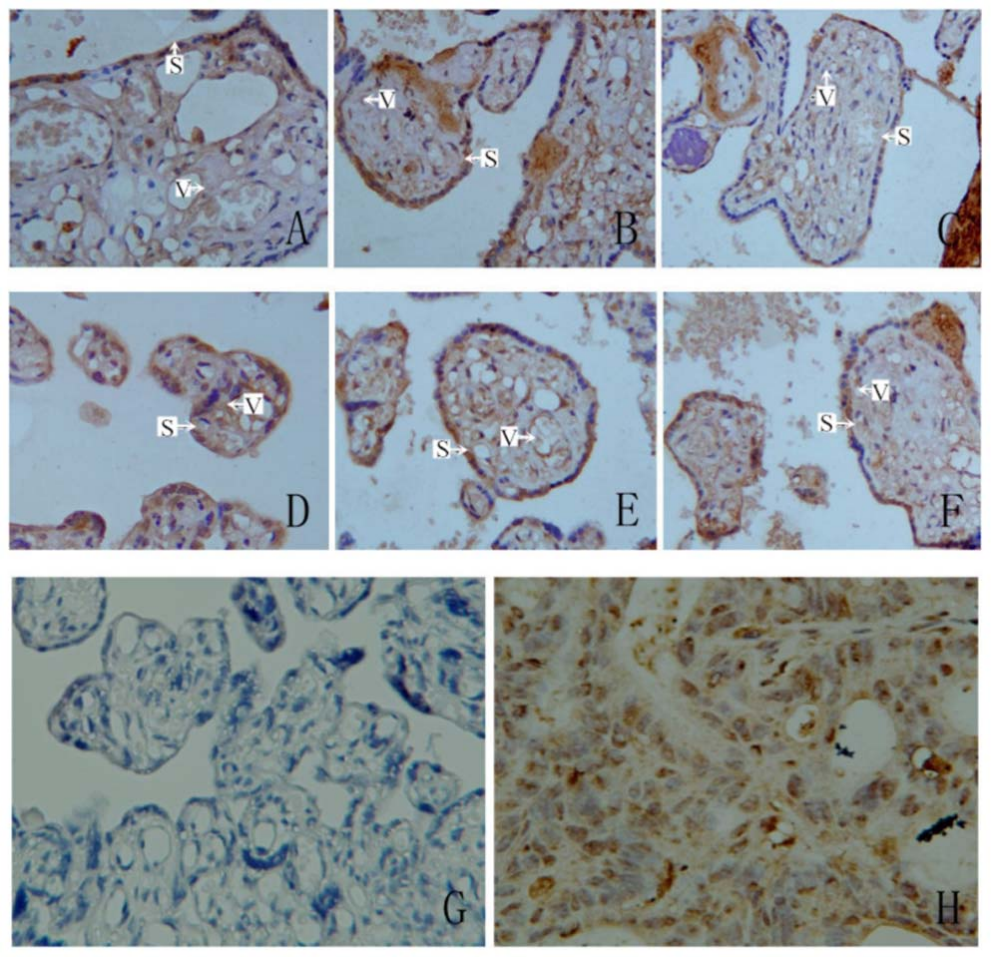
CXCL3 expression in placentae of three groups (Immunohistochemistry, SP, ×400). (S: syncytiotrophoblast; V: vascular endothelial cells) A: CXCL3 expressed on maternal surface of normal placentae; B: CXCL3 expressed on maternal surface of mild preeclamptic placentae; C: CXCL3 expressed on maternal surface of severe preeclamptic placentae; D: CXCL3 expressed on fetal surface of normal placentae; E: CXCL3 expressed on fetal surface of mild preeclamptic placentae; F: CXCL3 expressed on fetal surface of severe preeclamptic placentae; G: negative control; H: human colon adenocarcinoma tissue as positive control.

Placenta showed marked CXCL3 levels in trophoblast cells and vascular endothelial cells (both maternal and fetal sides) in all three groups. Human colon adenocarcinoma, which was used as the positive control sample, also expressed CXCL3 in the cytoplasm, whereas the negative control sample showed no immunochemical reaction (Fig. 2).

**Figure 3:**
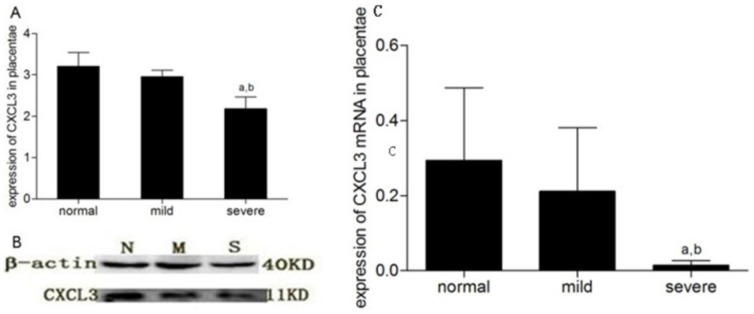
Placenta expression of CXCL3 and CXCL3-mRNA in normal pregnant women, mild preeclamptic pregnant women and severe preeclamptic pregnant women. A: Placenta CXCL3 was detected by western blotting (n = 6), Bar: mean; Whisker: standard deviation. a: p<0.01, versus normal pregnant women; b: p<0.01, versus mild pregnant women. B: N: normal; M: mild; S: severe. The experiment shown is representative of 1 of 6 performed. C: CXCL3-mRNA in each group (n = 12). Bar: mean; Whisker: standard deviation. a: p<0.05, versus normal pregnant women; b:p<0.05 versus mild pregnant women.

Semi-quantitative analysis was performed via Western blot after CXCL3 location was confirmed in the placenta. Protein levels were normalized to β-actin, and comparison among groups revealed that placental CXCL3 expression in the severe PE group (2.18±0.28) was significantly lower than that in the normal (3.21±0.34) and mild PE groups (2.96±0.15) (P<0.05); by contrast, no significant difference was noted between the normal and mild PE groups (P>0.05) (Fig. 3A). No relationship was observed between placental CXCL3 expression and clinical indicators (such as antepartum systolic and diastolic pressure, 24 h urine protein level, and liver and kidney function).

We conducted real-time fluorescent quantitative PCR to display the difference among groups with placental homogenate. mRNA levels were normalized to the house-keeping gene GAPDH by 2^-ΔΔCt^ [19] method. By comparison, we concluded that CXCL3-mRNA expression level in the severe PE group (0.014±0.012) was significantly lower than that in the mild PE group (0.211±0.17) and normal pregnant group (0.29±0.19, P<0.01), however there was no significant difference of CXCL3-mRNA level between normal and mild PE group (P >0.05, Fig. 3B).

### Viability and apoptosis of trophoblastic cell

**Figure 4:**
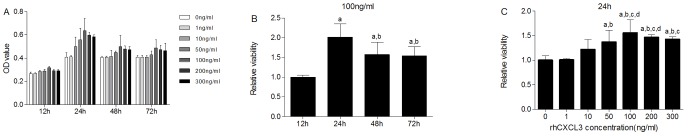
Effect of rhCXCL3 on the viability of trophoblast cells. Bar: mean; Whisker: standard deviation. A: HTR-8/SVneo cell viability in different rhCXCL3 concentration. B: Cell viability at different time intervals (12, 24, 48, and 72 h) . a: p<0.05, versus 12 h; b: p<0.05, versus 24 h. C: Cell viability in different rhCXCL3 concentration. a: a: p<0.05, versus 0 ng/ml; b: p<0.05, versus 1 ng/ml; c: p<0.05, versus 10 ng/ml; d: p<0.05, versus 50 ng/ml.

We performed a modified MTT assay (WST-1 assay) to investigate the effect of rhCXCL3 on the viability of early-pregnancy trophoblast cell line (HTR-8/SVneo cell). Treatment with rhCXCL3 at concentrations ranging from 0 ng/mL to 300 ng/mL enhanced HTR-8/SVneo cell viability in a dose- and time-dependent manner. Moreover, the maximal effect was induced by 100 ng/mL rhCXCL3 for 24 h. Higher rhCXCL3 concentrations did not result in a further increase in viability (Fig. 4). Fluorescence staining was then conducted to reveal the annexin V combination on trophoblast cells. rhCXCL3 decreased HTR-8/SVneo cell apoptosis in a concentration-dependent manner; however, this down regulation was insignificant (Figure S1).

### Invasion ability of trophoblastic cell

**Figure 5:**
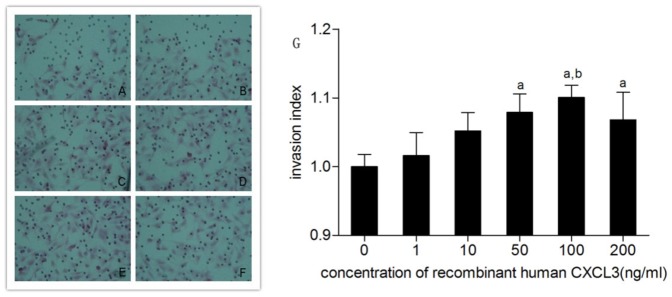
A: Invasion trophoblast cells stained by eosin under microscope (×400). A: 0 ng/ml rhCXCL3; B: 1 ng/ml rhCXCL3; C: 10 ng/ml rhCXCL3; D: 50 ng/ml rhCXCL3; E: 100 ng/ml rhCXCL3; F: 200 ng/ml rhCXCL3. G: Effect of rhCXCL3 on the invasiveness of trophoblast cells. Bar: mean; Whisker: standard deviation. a: p<0.05, versus 0 ng/ml; b: p<0.05, versus 1 ng/ml.

After the cells were treated with rhCXCL3 for 48 h, HTR-8/SVneo trophoblastic cell invasion gradually increased with increasing rhCXCL3 concentration. In addition, the highest invasion index was noted in 100 ng/mL rhCXCL3. The cells shaped as spindles and were stained red by eosin after 24 h of culture because of the carbonate membrane and adherent growth (Fig. 5A). Furthermore, the invasion indexes (invasion index  =  average cell number of one insert/average cell number of control insert) of each group revealed that rhCXCL3 promoted trophoblast cell invasion in a concentration-dependent manner (Fig. 5B).

## Discussion

A number of chemokines and their receptors have been identified at the maternal–fetal interface recently. They have been suggested to have different expression patterns based on their function at the human maternal–fetal interface [20], and dysregulationof this expression pattern will lead to preeclamptic onset [21], [22], [23]. Numerous researches have demonstrated that various chemokines were abnormal in preeclamptic patients [21], [22], [23], [24]. For instance, the plasma levels of CXCL8(IL-8), CCL2(MCP-1), CXCL10(IP10), and CXCL12(SDF-1) were elevated in severe preeclamptic women compared with those in normal pregnant women [21], [22], [24]. Besides, mRNA expression and quantification of CXCL12 in preeclampsia placenta were higher compared with those in normal placenta, especially in syncytiotrophoblasts of preeclampsia placenta [24], [25]. Furthermore, umbilical artery chemokine CCL16 and CCL24 levels were significantly higher in preeclampsia cords [23]. The main mechanisms of preeclampsia actually included altered angiogenic balance, immunological intolerance, inflammation/oxidative stress, and placental ischemia/hypoxia as a consequence or cause of shallow cytotrophoblast invasion and incomplete remodeling of uterine spiral arteries [26]. In addition, chemokines can regulate the immunity microenvironment in fetal–maternal interface, which is beneficial to embryonic implantation and development; by constrast, failure of this process to proceed can lead to preeclampsia [27]. Studies on chemokine mechanism that resulted in preeclampsia are conducted to investigate its effect on neovascularization, trophoblast cell invasion, and fetal–maternal interface immunity microenvironment [15], [28], [29].

ELR+ CXC chemokines can promote migration and viability of endothelial cells and are potent neovascularization promoters. The proangiogenic property of ELR+ CXC chemokines has been demonstrated in many pathological processes. In a myocardial acute ischemia model, increased CXCL1 and CXCL8 have central function in regulating vasculogenesis [30]; chemokines (CXCL1, CXCL2, CXCL3, CXCL5, and CXCL7) secreted by monocytes in vitro culture were able to induce microvessel formation [31]. On the one hand, CXCL3 had a significant developing gene in colon carcinoma and a significantly down-regulated expression level in both lymph node metastasis and distant metastasis [32], and high expression of CXCL3 in breast cancer cells was relevant with tumor metastasis [33]. On the other hand, recombinant human CXCL3 can induce airway smooth muscle cell migration [34]. In the present study, the CXCL3 expression was significantly lower in the placenta of preeclamptic women compared with that of normal women. In addition, relativit**y** between CXCL3 expression and important clinical indexes in severe preeclampsia suggested that CXCL3 expression level was related to preeclampsia severity. As an ELR+ CXC chemokine, CXCL3 was chemotactic for neutrophils and pro-angiogenic subtances [29]; therefore, a low CXCL3 expression in preeclamptic placenta may contribute to maternal endothelial dysfunction and incomplete remodeling of uterine spiral arteries during placentation, which need further experiments confirmed. Although mechanism exploration was not conducted, Laila demonstrated that CXCL3-induced migration was dependent on p38 and ERK1/2 MAPK pathways via CXCR1 and CXCR2 [34].

The abnormal expression of chemokine was believed to regulate trophoblast invasion by affecting the biological behavior of trophoblast cells. For instance, CXCL8 (IL8) can promote trophoblast cell migrate and invade into the endometrium in the first trimester by enhancing MMP-2 and MMP-9 expression [35]. Trophoblast cell-derived chemokine CXCL12 enhanced trophoblast cell invasiveness through both autocrine-enhancing expression of MMP and paracrine-increasing CD82 expression in endometrial stromal cells [36], and exogenous CXCL16 significantly improved trophoblast invasiveness and viability in vitro experiments [37]. We examined the invasive capacity of HTR-8/SVneo cells to reveal if exogenous CXCL3 had an analogous effect. The results indicated that recombined human CXCL3 remarkably strengthened trophoblast cell invasion in line with previous studies. Similarly, our results showed that recombination of human CXCL3 enhanced trophoblast viability, which might be synergistic with invasiveness. CXCR2,a key receptor involved neovascularization, was the exclusive receptor of CXCL3. CXCR2 was found in endothelial cells and induced the migration, invasion, and viability of the endothelial cells, which subsequently resulted in angiogenesis. CXCL3 has a function in the process, although this chemokine was not involved in this study. The cytotrophoblast expression of CXCR2 was demonstrated at the transcriptional level [38]. This process led to possible CXCL3 binding, which confirmed the function of CXCL3 in CXCR2 cytotrophoblast expression.

Our results showed that plasma CXCL3 levels increased, whereas placental CXCL3 expression decreased in the preeclampsia group. This incongruity was considered to be a compensatory mechanism. We considered that low expression of CXCL3 at placenta site resulted in shallow implantation, as a main reason for the onset of preeclampsia. However, the source of increased CXCL3 in preeclampsia plasma was not clear, which mayact as compensatory role during pathogenesis in other parts. Various compensatory mechanisms were proposed, such as neovascularization or angiogenesis, to conquer uteroplacental hypoxia in preeclampsia. However, these mechanisms have been poorly examined to date. Manifestations of symptoms, such as hypertension, proteinuria, and edema, may be explained if the compensatory mechanisms of preeclampsia were understood.

## Supporting Information

Figure S1 **Effect of rhCXCL3 on annexin V combination of trophoblast cells.**
(TIF)
