# Supplementary figures and images for: Inconformity of CXCL3 Plasma Level and Placenta Expression in Preeclampsia and Its Effect on Trophoblast Viability and Invasion
Source: PLoS One. 2014 Dec 8;9(12):e114408. doi: 10.1371/journal.pone.0114408 (PMC4259324; doi:10.1371/journal.pone.0114408)

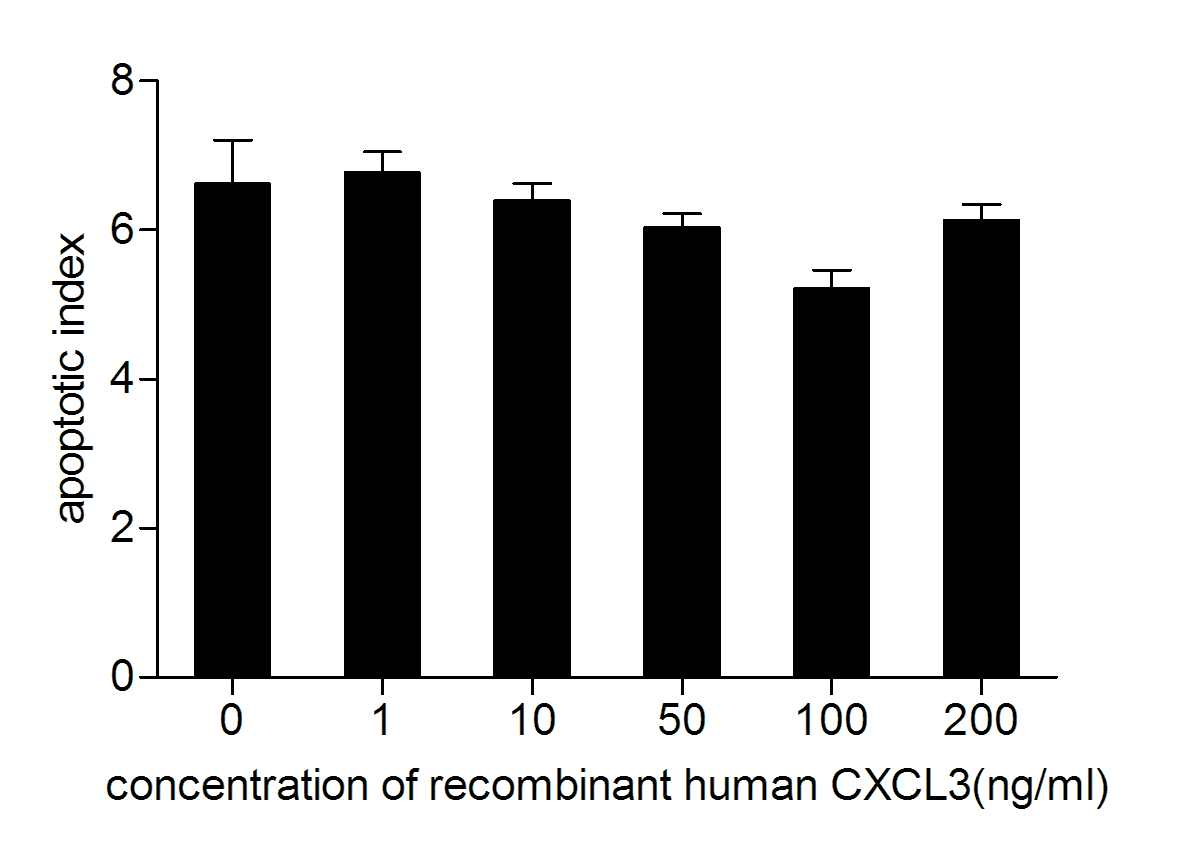

Supplement: Figure S1 — Effect of rhCXCL3 on annexin V combination of trophoblast cells. (TIF) [file pone.0114408.s001.tif]
